# Experimental Study on Influence of Lime on Cross-Scale Characteristics of Cemented Backfill with Multiple Solid Wastes

**DOI:** 10.3390/ma17164090

**Published:** 2024-08-17

**Authors:** Xiaosheng Liu, Weijun Wang, Zhengwei Han

**Affiliations:** 1 School of Resources, Environment and Safety Engineering, Hunan University of Science and Technology, Xiangtan 411201, China; 1010109@hnust.edu.cn; 2 School of Minerals Processing and Bioengineering, Central South University, Changsha 410083, China

**Keywords:** cemented backfill, industrial solid wastes, hydration mechanism, macro and meso characteristics, cross-scale functional relationship

## Abstract

The utilization of industrial solid waste in mines is an important approach to resource utilization. The backfill material in mines is mainly composed of solid waste, which plays a supporting role. The excitation effect of lime on phosphogypsum and fly ash in backfill was studied in this paper. The macroscopic and microscopic characteristics of the backfill material were tested using uniaxial compression, nuclear magnetic resonance, scanning electron microscopy, and electrochemical techniques, and a relationship model was established between them. Furthermore, the influence of industrial solid waste on the properties of the backfill material under the action of lime and the hydration mechanism between different industrial solid wastes were studied. The results show that (1) under the action of lime, fly ash reacts with lime to produce C-S-H and C-A-H, and then C-A-H reacts with phosphogypsum to produce AFt. (2) The excess phosphogypsum also fills the pores. Therefore, 1.8% lime reduces the porosity of the backfill by 17.88% and increases the strength by 21.57%. (3) The cross-scale relationship shows that strength is inversely proportional to each type of pore content and fractal dimension, and it logarithmically increases with impedance at different frequencies. The lower the frequency, the stronger the relationship is. (4) This study indicates that industrial solid waste is a suitable cement replacement.

## 1. Introduction

The treatment of industrial solid waste is an urgent problem that enterprises need to solve [1,2]. Filling the goaf with backfill material can provide effective support, which can not only prevent surface collapse but also improve the ore recovery rate and ensure the safety of the stope [2,3]. However, cement that plays a binding role in the backfill material not only increases the filling cost but also pollutes the environment during its production. Therefore, searching for cement substitutes is a hot research topic. Generally, solid wastes with potential activity are sought instead, such as steel slag (SS), fly ash (FA), phosphogypsum (PG), etc. [4,5,6]. Studies have shown that the use of physical excitation or chemical excitation (alkali, salt materials) can stimulate their gelling properties, enabling them to replace cement [7,8,9].

PG, as a waste produced in the phosphorus chemical production process, is mainly composed of calcium sulfate, with a few other oxides and impurities [10]. Some studies have shown that PG can be used as an aggregate for backfill material, but its composition, of impurities, is harmful to the performance of backfill. PG after pretreatment can effectively improve various properties of backfill [11,12]. A study has found that the strength of pretreated PG as an aggregate is eight times higher than that of untreated PG as an aggregate [12]. Some studies have also shown that PG has certain potential activity, which can be activated by alkaline materials (such as lime, calcium carbide slag, etc.) [13,14]. Guanzhao Jiang et al. found that lime can improve the early strength of PG as a filling binder [14]. Fly ash (FA) is a solid waste discharged from coal-fired plants, and its main components are SiO_2_ and Al_2_O_3_ [15]. It has a similar composition to cement and has certain properties. Some scholars have added FA to backfill materials and found that an appropriate amount of FA can effectively improve the strength of backfill, but excessive addition is harmful to the strength of backfill [16]. Therefore, FA can be used instead of cement. Some studies have shown that FA can be used to replace a portion of cement under the condition of ensuring the strength requirements of mine backfill, indicating that FA is a suitable cement replacement [17]. Studies have shown that under the action of lime activation, FA can undergo a pozzolanic reaction, which promotes the strength of the backfill [9]. According to the hydration reaction mechanism [18], the combined action of lime, PG, and FA can produce a better hydration effect. Therefore, these three materials can be used to make gelling materials. A scholar has found that a blended binder composed of 4.76% lime, 71.43% FA, and 23.81% PG can play an effective gelling role, similar to that of Portland cement [19]. However, there are a few studies on the simultaneous activation of both FA and PG by lime, so it is necessary to further examine this process. Thus, studying the replacement of mine cement is essential to reduce mine filling costs.

Based on this and the shortcomings of the above studies, this study prepared backfill materials by using crushed stone, FA, PG, lime, and cement as raw materials. Uniaxial compression, nuclear magnetic resonance (NMR), scanning electron microscopy (SEM), and electrochemical technology were used to test the macro and micro characteristics of backfill. A cross-scale function relationship was established, and a comprehensive analysis was conducted to evaluate the influence of PG and FA on the macro and micro characteristics of the backfill under the action of lime, as well as the mechanism of action between the three kinds of solid wastes.

## 2. Scheme Design

### 2.1. Raw Materials

**Figure 1:**
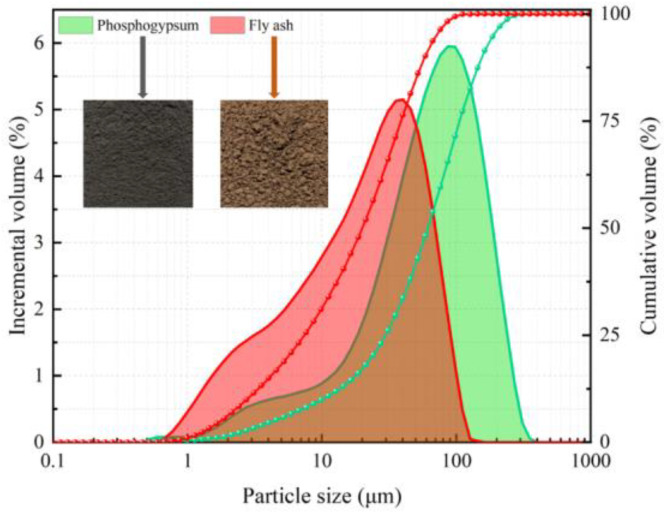
Particle size distribution of PG and FA.

The PG and FA came from a chemical plant in Yichang, Hubei. The crushed stone came from a mine in Yichang, Hubei. A laser particle size analyzer and XRF were used to test the particle size and composition of the PG and FA. Their particle size distribution is shown in Figure 1, and their chemical composition is shown in Table 1. It can be seen from Table 1 that the CaSO_4_ content in PG is 89.63%, and the Al_2_O_3_ content in FA is 30.56%. The particle size of the crushed stone was analyzed by the screening method, and its particle size distribution is shown in Table 2. It can be seen from Table 2 that the particle size of the crushed stone is relatively large. The lime used in this study, purchased online, contains 85% CaO. The water used in the experiment was Changsha drinking water. The cement was commercially available ordinary Portland cement.

### 2.2. Experimental Method

**Figure 2:**
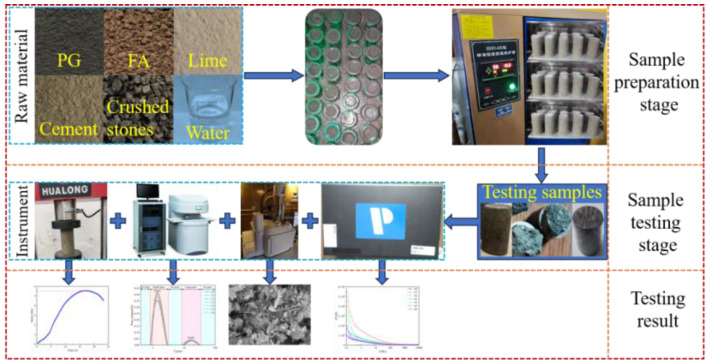
Experimental flowchart.

In this experiment, crushed stone was used as the aggregate, cement was used as the primary cementitious material, and phosphogypsum, fly ash, and lime were used as new cementitious materials. The sample preparation process involved first weighing the raw materials according to the ratios (Table 3) to prepare a slurry, then injecting the slurry into a standard cylindrical mold (φ 50 × 100 mm), and marking each group of samples as A0–A6 according to the ratio. After the slurry hardened, the samples were demolded and placed in a curing box for curing. Various tests were then conducted on the 7th day of curing. The experimental process is shown in Figure 2.

The testing section included four parts. The first part was strength testing, with three samples taken from each group. The instrument model was WHY-300/10. The testing parameter was a loading rate of 0.2 kN/s. The second part was SEM testing, where the sample came from the broken fragments (about 1 cm^2^) that were tested in the first part. The instrument model was Tescan Mira3. The fragments were processed (drying and gold spraying) and placed in the instrument for testing. The third part was NMR testing, where the sample was saturated with water. The instrument model was MesoMR23-060H. After adjusting the parameters, the whole sample was put into the instrument for testing. The fourth part was electrochemical testing. The samples used in this part were the same as those used in the NMR testing. The conductive sheets were placed on the upper and lower surfaces of the samples, and parameters were set for testing.

## 3. Results

### 3.1. UCS Attribute

**Figure 3:**
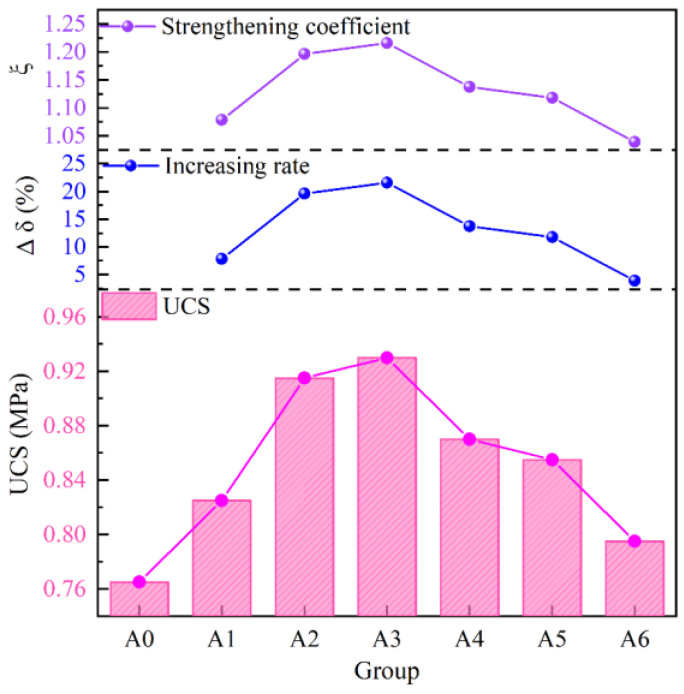
UCS of all groups.

Strength is the most basic attribute of backfill. Since backfill mainly plays a supporting role, uniaxial compressive strength (UCS) is the most important attribute. A UCS test was performed on the sample, and the results are shown in Figure 3. It can be seen from Figure 3 that the strength increases initially and then decreases with the increase in lime content. In order to further analyze the influence of lime on strength, two parameters *ξ* and Δ*δ* are introduced, with their calculation formulas as follows:

(1) $$\xi =\frac{\sigma_{i}}{\sigma_{0}}$$

(2) $$\Delta \delta =\frac{\sigma_{i}-\sigma_{0}}{\sigma_{0}}\times 100\%$$

where *ξ* represents the strength strengthening coefficient, Δ*δ* represents the strength growth rate (%), *σ_i_* represents the strength of each group (MPa), and *σ_0_* represents the strength of group A0 (MPa). When *ξ* is greater than 1 and Δ*δ* is greater than 0, the strength has a gain effect. It can be seen from Figure 3 that ξ is greater than 1 and Δ*δ* is greater than 0, indicating that lime has a gain effect on strength. The lime reacts with the FA and PG to produce more hydration products, thus increasing the strength of the backfill. The *ξ* and Δ*δ* values in group A3 are the highest, at 1.22 and 21.57%, respectively, indicating that this group has the best lime content.

### 3.2. NMR Characteristic

**Figure 4:**
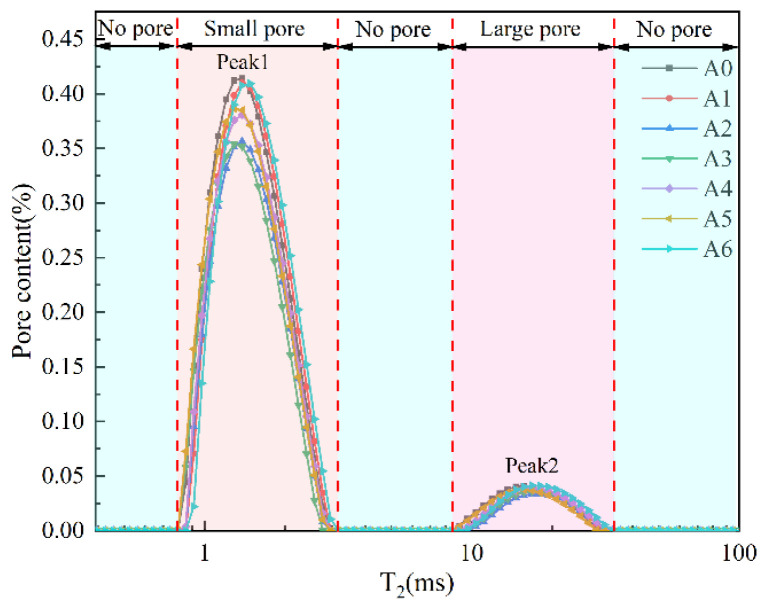
NMR characteristics of all groups.

Pore characteristics are an important feature affecting the strength of backfill. The pore distribution of the whole sample can be nondestructively detected by NMR. The samples of each group were tested by NMR, and the results are shown in Figure 4. As can be seen from Figure 4, the NMR spectrum has two peaks, which represent different pores. T_2_ is the transverse relaxation time, which is a time characteristic parameter of NMR spectra. According to relevant studies [20,21], there is a direct proportional relationship between pore size and T_2_ value. The larger the T_2_ value, the larger the pore size. Therefore, in Figure 4, peak 1 represents a small pore, and peak 2 represents a large pore. There are two types of pores in the backfill, most of which are small pores. The pore size of the large pore is approximately 10 times that of the small pore. The pore size of the large pore is not continuous with the pore size of the small pore. It can be seen from the peak distribution that there is a significant change in peak 1 and a small change in peak 2, which indicates that the addition of lime has a significant impact on small pores while having little effect on large pores.

**Figure 5:**
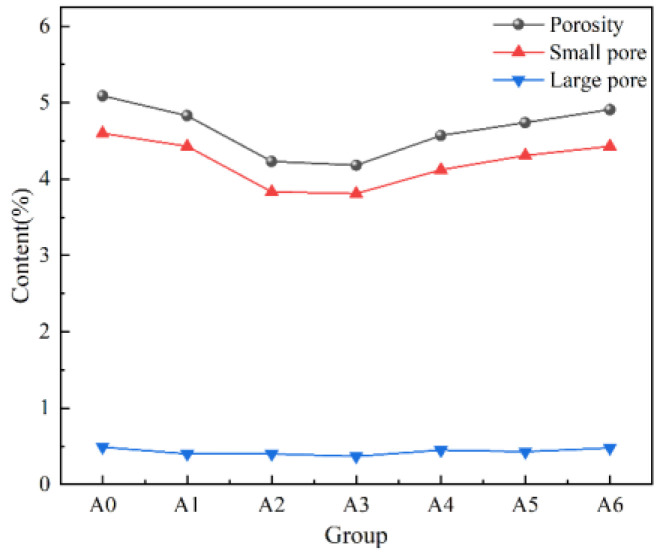
Pore content of various types of pores.

The pore content of NMR is statistically analyzed, and the results are shown in Figure 5. It can be seen from Figure 5 that with the increase in lime content, both the porosity and pore contents of the small pore and large pore show a trend of first decreasing and then increasing. The changes in porosity and small pore content are significant, while the changes in large pore content are more gradual. The pore content of group A3 was the lowest, with a lime content of 1.8%. This is because adding an appropriate amount of lime can optimize the hydration reaction of industrial solid waste, thereby producing the most hydration products and filling the pores inside the backfill, resulting in the smallest porosity.

### 3.3. SEM Analysis

**Figure 6:**
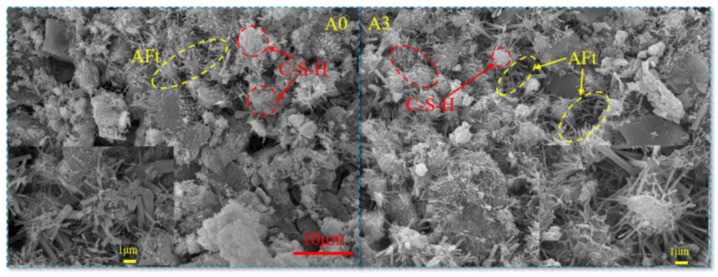
SEM images of backfill.

The classification of hydration products can be determined by analyzing their morphology in SEM images. Figure 6 shows the SEM images of the backfill without lime and with optimal lime content. As can be seen from Figure 6, the hydration products are mainly AFt crystals and C-S-H gels, which are intertwined, with some covered on the aggregate and others filling the pores. It can be seen from the SEM image that lime can increase the amount of hydration products, explaining the data about hydration products. A comparative analysis found that the hydration products increased significantly after adding lime. This is because PG contains a large amount of CaSO_4_ and a small amount of SiO_2_ and Al_2_O_3_. The main components of FA are SiO_2_ and Al_2_O_3_. The main component of lime is CaO, which forms Ca(OH)_2_ when it reacts with water, and there is also a small amount of Ca(OH)_2_ in the hydration products of cement. Therefore, reactions can occur between them under sufficient reactant conditions, and the reaction relationship is as follows [22,23,24]:

(3) $$Ca{\left(OH\right)}_{2}+SiO_{2}+H_{2}O\to 3CaO\cdot 2SiO_{2}\cdot {3H}_{2}O$$

(4) $$Ca{\left(OH\right)}_{2}+{Al}_{2}O_{3}+H_{2}O\to CaO\cdot {Al}_{2}O_{3}\cdot {6H}_{2}O$$

(5) $$3Ca{SO}_{4}\cdot 2H_{2}O+3CaO\cdot {Al}_{2}O_{3}\cdot 6H_{2}O+H_{2}O\to 3CaO\cdot {Al}_{2}O_{3}\cdot 3Ca{SO}_{4}\cdot 32H_{2}O$$

From the above reactions, it can be seen that after adding lime, FA undergoes a reaction to form C-S-H and C-A-H, and then C-A-H reacts with PG to form AFt. Within a certain range, the more lime is added, the higher the content of hydration products, which optimizes the performance of the backfill material and is also the reason for the increase in strength of the backfill material.

### 3.4. AC Impedance Characteristic

Impedance is an indicator of the ability of a circuit to obstruct the passage of current, and its value is related to the frequency of the current. The impedance of different substances also varies, and if the composition of the same substance is different, its impedance is also different. Impedance can therefore be used to study the internal composition of substances.

**Figure 7:**
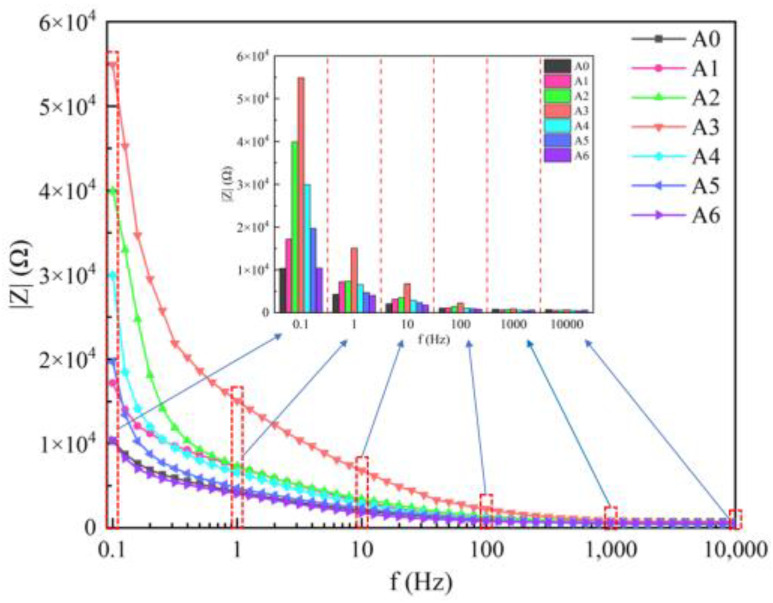
The impedance values of all groups at different frequencies.

The backfill was tested with an electrochemical instrument, and the results are shown in Figure 7. As can be seen from Figure 7, with an increase in frequency, the impedance value first decreases sharply and then stabilizes. When the frequency reaches 100 Hz, the impedance value basically remains unchanged, which shows that the impedance change of the backfill is most noticeable at a low frequency. Since the backfill is similar to concrete, the results are similar to those of Biqin Dong et al. on concrete [25]. From group A0 to group A6, as the lime content increases, the impedance first increases and then decreases.

## 4. Discussion and Analysis

### 4.1. Pore Characteristics Analysis

**Figure 8:**
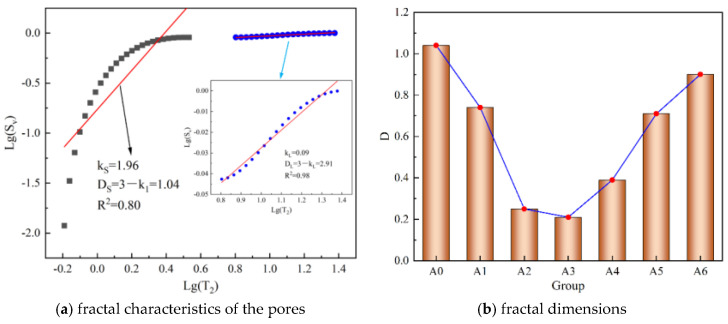
Fractal results of backfill.

The commonly used method for quantitative analysis of pore characteristics is fractal analysis. Fractal characteristics can reflect the complexity of pores, that is, the larger the fractal dimension, the larger and more complex the pores. According to relevant studies [20,21], the fractal method based on NMR can be expressed in the form of Equation (6). The fractal characteristics of the pores in the backfill can be calculated using this fractal method, as shown in Figure 8a (group A0 as example), and the fractal dimensions of each group are shown in Figure 8b. It can be seen from Figure 8a that the larger the pore size, the larger the fractal dimension, indicating that the pores are more complex. It can be seen from Figure 8b that the fractal dimension is between 0.21 and 1.04 and first decreases and then increases with the increase in lime content, indicating that a suitable lime content can reduce the pore size and make the pores more regular.

(6) $$\mathrm{lg}\left(S_{v}\right)=\left(3-D\right)\mathrm{lg}\left(T_{2}\right)+\left(D-3\right)lg(T_{2max})$$

where *S_v_* represents the cumulative amount of pores (%), and *D* represents the fractal dimension. It can be seen from Equation (6) that there is a linear relationship between Lg(*S_v_*) and Lg(*T_2_*), and the sum of the slope k and the fractal dimension *D* is 3. Therefore, *D* can be calculated by k (D = 3 − k).

### 4.2. The Relationship between UCS and Pore Parameters

#### 4.2.1. The Relationship between UCS and Pore Content

**Figure 9:**
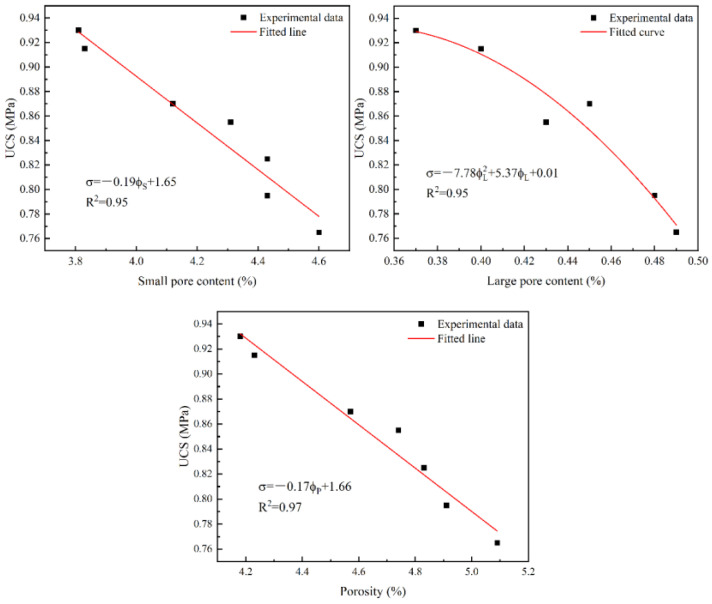
The relationship between UCS and pore content.

Hydration products have a certain filling effect on backfill pores. Therefore, industrial solid waste under the action of lime affects the pore content of backfill, thus changing the strength of backfill. The functional relationships between backfill strength and pore content have been established, and the results are shown in Figure 9. As can be seen from Figure 9, the fitting effect between them is very good (R^2^ > 0.95). They conform to the following relationship:

(7) $$\sigma =-0.19\phi_{S}+1.65$$

(8) $$\sigma =-7.78\phi_{L}^{2}+5.37\phi +0.01$$

(9) $$\sigma =-0.17\phi_{P}+1.66$$

where *σ* represents UCS (MPa), ϕ*_S_* represents small pore content (%), ϕ*_L_* represents large pore content (%), and ϕ*_P_* represents porosity (%). The strength of backfill decreases with the increase in various types of pore content, indicating that the higher the pore content, the lower the strength. The decline law is different for different pore types. For large pores, the greater the pore content, the greater the strength reduction rate, indicating that large pores have a greater impact on strength. Since small pores have more content, the changing law of strength with small pore content is similar to that of porosity.

#### 4.2.2. The Relationship between UCS and Fractal Dimension

**Figure 10:**
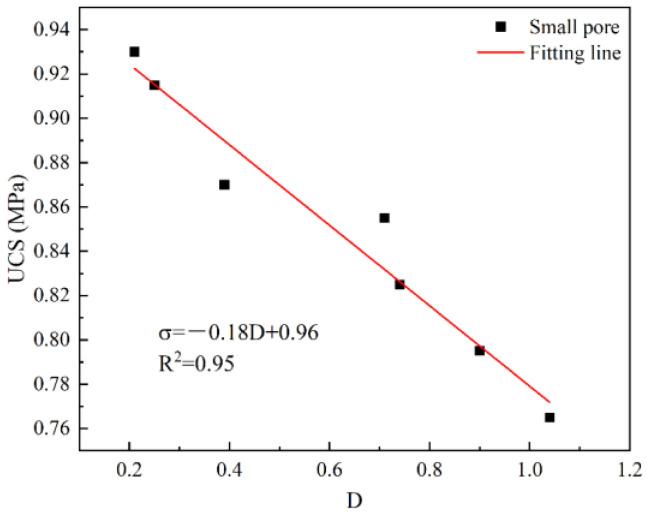
The relationship between UCS and fractal dimension.

Hydration products have a certain impact on pores, and the fractal dimension reflects the complexity of pores; therefore, it also has a certain impact on strength. The relationship between strength and fractal dimension has been established, and the results are shown in Figure 10. It can be seen from Figure 10 that there is a good relationship between intensity and fractal dimension (R^2^ = 0.95), which follows a decreasing relationship and can be expressed as Equation (10). It can be seen from Equation (10) that the strength decreases with the increase in the fractal dimension; that is, the larger the aperture, the more complex the pores and the lower the strength. This is consistent with the research results of some scholars [21].

σ = −0.18 D + 0.96 (10)

### 4.3. The Influence Law of Liquid Content on Impedance

**Figure 11:**
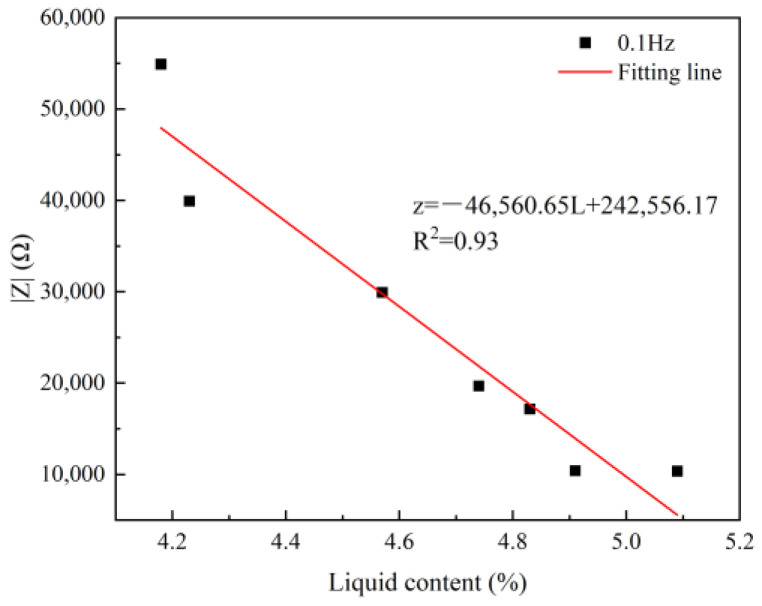
The relationship between liquid content and impedance.

The liquid content in the backfill also has a certain impact on impedance. The testing samples of NMR and electrochemistry are the same sample, and both are saturated water samples, so the liquid content measured by NMR can be used for electrochemistry. The relationship between impedance and liquid content has been established, and the result is shown in Figure 11. As can be seen from Figure 11, there is a good relationship between impedance and liquid content (R^2^ = 0.93), and they conform to the following relationship:

z = −46,560.65 L + 242,556.17 (11)

where L represents the liquid content, %. As the liquid content increases, the impedance value decreases linearly, which indicates that the liquid has good conductivity, so the higher the liquid content, the smaller the impedance.

### 4.4. The Influence Law of Impedance on UCS

**Figure 12:**
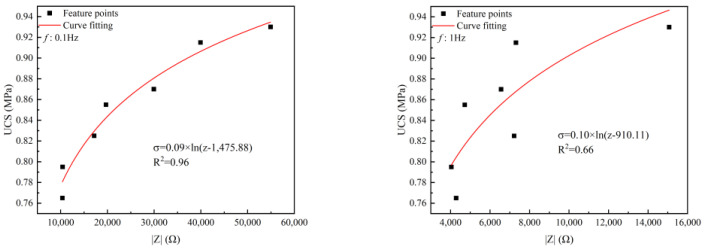

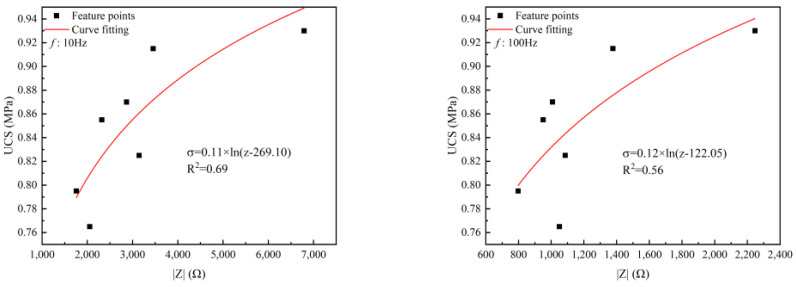
The relationship between UCS and impedance at different frequencies.

The change in the impedance value reflects the change in the internal structure of the backfill, and the influencing factors of the strength can be analyzed from the microscopic point of view. By establishing the relationship between the strength and the impedance under different frequencies, the influence of the internal structure on the strength is analyzed. The relationship under different frequencies was obtained by fitting the data, as shown in Figure 12. It can be seen from the fitting effect that the higher the frequency, the worse the effect, and the best effect is at 0.1 Hz (R^2^ = 0.96). When the frequency reaches 100 Hz, the fitting effect is very poor (R^2^ = 0.56). When the frequency ranges from 0.1 to 100 Hz, they all conform to the logarithmic relationship (that is, y = a*ln(x − b)), which is similar to the results of Wenbin Xu et al. [26]. Under 0.1–100 Hz, the functional relationship is as follows:

At 0.1 Hz

(12) σ=0.09×ln⁡(z−1475.88)

At 1 Hz

(13) σ=0.10×ln⁡(z−910.11)

At 10 Hz

(14) σ=0.11×ln⁡(z−269.10)

At 100 Hz

(15) σ=0.12×ln⁡(z−122.05)

where z represents impedance (Ω). The UCS increases logarithmically with the increase in impedance. This is because the backfill mainly includes three conductive phases (namely, solid, liquid, and gas), and the connection combination between them is solid–liquid-gas, solid–liquid, solid–gas, and solid–solid. The conductivity of different phases varies and is generally liquid > solid > gas. When the impedance is large, it indicates that the conductive effect is poor, so there is more solid or gas. Since the gas volume in the backfill is less, the density of the backfill is better and the UCS is higher.

## 5. Conclusions

This paper studies the influence of lime on the macro and micro characteristics of backfill material with industrial solid waste, as well as the hydration mechanism between industrial solid waste. It can provide guidance for the application of industrial solid waste in mines and find suitable replacements for cement. The main conclusions are as follows:

(1) Under the action of lime, FA undergoes a pozzolash reaction to produce C-S-H gelling and C-A-H crystals. C-A-H crystals then react with PG to produce AFt, which is similar to the hydration reaction of cement.
(2) The industrial solid waste under the action of lime has an optimized strength effect. The addition of 1.8% lime can increase the strength by 21.57%, indicating that the hydration product of industrial solid waste has a filling effect, resulting in a 17.88% reduction in the backfill porosity. The hydration product also has a bonding effect, so the strength is increased.
(3) Fractal characteristics reflect the complexity of pores. The larger the pore size, the larger the fractal dimension, indicating that the pores are more complex. The fractal dimension is between 0.21 and 1.04 and first decreases and then increases with the increase in lime content.
(4) The cross-scale relationship of strength is established. The results show that strength is inversely proportional to various types of pore contents and fractal dimensions and increases logarithmically with impedance at different frequencies. The lower the frequency, the stronger the relationship is.

## Figures and Tables

**Table 1:** The elemental composition of PG and FA.

Element	O	Ca	Si	Al	Fe	Mg	S	F	Other
PG	36.96	35.07	4.27	0.41	0.57	0.14	21.09	0.33	1.16
FA	33.89	5.89	28.24	16.18	8.11	0.80	1.38	0.27	5.24

**Table 2:** Particle size distribution of crushed stones.

Particle Size/mm	Mass Fraction/%	Mass Accumulation/%
0.00−0.28	25.00	25.00
0.28−1.25	19.50	44.50
1.25−2.00	20.25	64.75
2.00−3.00	15.00	79.75
3.00−4.00	12.75	92.50
4.00−5.00	7.50	100

**Table 3:** Experimental proportion.

Group	Lime/PG (%)	PG Content (%)	FA Content (%)	Mass Percentage	Cement–Crushed Stones Ratio
A0	0	20	5.9	80%	1:8
A1	0.2				
A2	1				
A3	1.8				
A4	2.6				
A5	3.4				
A6	4.2				

## Data Availability

The datasets used and/or analyzed during the current study available from the corresponding author on reasonable request.
